# Interface engineering through electron transport layer modification for high efficiency organic solar cells†

**DOI:** 10.1039/c7ra13428b

**Published:** 2018-02-05

**Authors:** Kunal Borse, Ramakant Sharma, Dipti Gupta, Aswani Yella

**Affiliations:** Department of Metallurgical Engineering and Materials Science, Indian Institute of Technology Bombay Powai Mumbai-400076 India diptig@iitb.ac.in aswani.yella@iitb.ac.in; Department of Metallurgy, Government Polytechnic Kolhapur-416004 India

## Abstract

In the present study, we have compared the device performance of poly[4,8-bis(5-(2-ethylhexyl)thiophen-2-yl)benzo[1,2-*b*;4,5-*b*′]dithiophene-2,6-diyl-*alt*-(4-(2-ethylhexyl)-3-fluorothieno[3,4-*b*]thio-phene-)-2-carb-oxylate-2-6-diyl)] (PTB7-Th):phenyl-C71-butyric acid methyl ester (PCBM) organic solar cells (OSCs) in an inverted geometry with ZnO, a bilayer of ZnO and Ba(OH)_2_ [ZnO/Ba(OH)_2_] and a nanocomposite of ZnO and Ba(OH)_2_ [ZnO:Ba(OH)_2_] as electron transport layers (ETLs). Our study reveals that the performance of the devices with the ZnO/Ba(OH)_2_ and ZnO:Ba(OH)_2_ nanocomposite as ETL supersedes that of devices with only ZnO as ETL. The plausible reasons for the improved performance of these devices are identified using morphological studies, contact angle measurements, X-ray photoelectron spectroscopy (XPS), ultraviolet photoelectron spectroscopy (UPS) and photo-electrochemical impedance spectroscopy (EIS) measurements. It is observed that films of ZnO/Ba(OH)_2_ and ZnO:Ba(OH)_2_ nanocomposites have a low work function and are slightly more smooth and hydrophobic than ZnO films. This might have suppressed the charge recombination and thereby improved the charge collection as has been confirmed by EIS measurements.

## Abstract 2

Schematic of PTB7-Th:PCBM OSCs in an inverted geometry with ZnO, ZnO/Ba(OH)_2_ and ZnO:Ba(OH)_2_ nanocomposites as ETLs.

## Graphical abstract

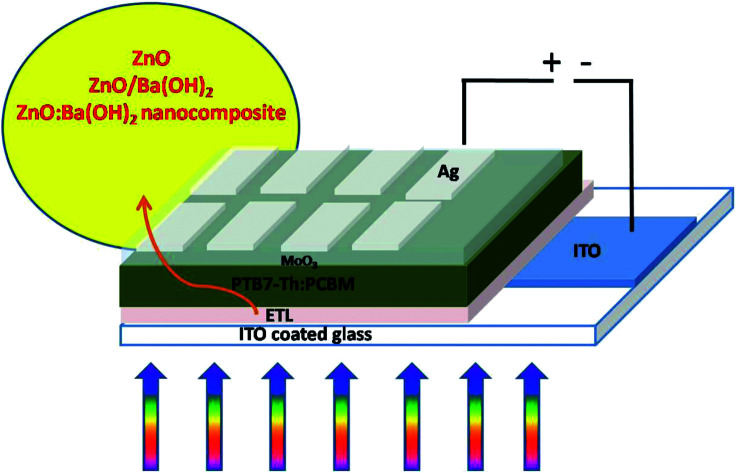

## 1. Introduction

Solution processed bulk-heterojunction organic solar cells (OSCs), which consist of a conjugated polymer as an electron donor and fullerene and/or polymer as an acceptor, are widely regarded as an emerging PV technology and offer many advantages such as mechanical flexibility, low-cost, light weight, low energy consuming roll-to-roll manufacturing process, *etc.*^1–5^ Over the years, to increase the power conversion efficiency (PCE) of OSCs, various strategies have been used. This includes designing and developing new donor and/or acceptor molecules having wide absorption spectra and/or employing various aspects of device and interface engineering such as use of solvent additives for photoactive layer processing, incorporation of metals, dielectric and/or semiconducting nanoparticles (NPs) in the photoactive layer, use of buffer layers like electron and/or hole transport layers, *etc.*^6–12^ It has been demonstrated in the past that the careful selection of electron and/or hole transport layers can improve the PCE by 2–3% for the same donor:acceptor bulk-heterojunction system.^12–17^

Till date several types of material and/or their nanocomposites have been used as an electron transport layers (ETLs).^2,18–20^ Among them the most preferred ones are solution processed metal oxides (MeO_*x*_) like ZnO, TiO_2_, ZrO_2_, *etc.*^21–23^ This is quite obvious as most of these MeO_*x*_ offer good air-stability, high transparency to visible light, high electron affinity, and tuneable electrical and optical properties.^24,25^ However, being a solution processed and low temperature processed, these MeO_*x*_ often digress from the properties of single crystals and as a result, many-a-times, it is observed that defect related energy levels or Schottky barriers form interfacial barriers.^26^ Besides MeO_*x*_, organic dipole layers such as conjugated polyelectrolytes (CPE),^2,27–29^ organic salts, zwitterions and cost effective poly(ethyleneimine) (PEI)^19^ or poly-(ethyleneimine)-ethoxylated (PEIE)^20^ does modify the work function and has been used an ETL. Most of them can simply be spin-coated in ambient atmosphere just like MeO_*x*_ and helps in simplifying the fabrication method. However, the insulating nature of these types of materials often leads to high series resistance in a thick film and therefore the performance of devices was found to be highly sensitive to the thickness of these layers.^30–32^

To overcome these issues, in recent times, two alternate approaches which include use of bilayer structure such as MeO_*x*_/PEI (PEIE/PFN) and MeO_*x*_:PEI (PEIE/PFN) nanocomposite as an ETL have been studied.^19,20,33,34^ Although the bilayer structure improves the PCE of the devices, the high thickness sensitivity of performance caused by PEI (PEIE) still can't be solved.^30–32^ On the other hand, MeO_*x*_:PEI (PEIE/PFN) nanocomposite offers combined advantages such as the high charge carrier mobility of MeO_*x*_ and good film formation ability of polymer.^33–35^ As a result, devices with nanocomposite as an ETL often demonstrate high PCE and good stability. Recently, less-studied type of inorganic salt, barium hydroxide [Ba(OH)_2_] has been explored as an ETL so as to improve device performance in polymer light-emitting diodes (PLEDs),^36^ organic field effect transistors (OFETs),^37^ OSCs owing to its outstanding charge injection ability. A remarkable enhancement in PCE of OSCs has been demonstrated by employing Ba(OH)_2_ as an ETL or upon inserting a Ba(OH)_2_ layer between photoactive layer and ETLs.^26,38^ Both this approaches contribute to reduction of energetic barrier at the interface and improves charge collection.

Here in, we have adopted a novel approach in which we have blended a sol–gel ZnO solution with a Ba(OH)_2_ solution prior to their final consolidation into the nanocomposite layer as the ETL. From the similar type of previous studies, we presume that the presence of Ba(OH)_2_ in ZnO might induce stronger electrostatic or dipole–dipole interactions with a ZnO surface and therefore use of ZnO:Ba(OH)_2_ nanocomposites as ETLs would improve the device performance of OSCs. Devices were fabricated in an inverted geometry having a structure ITO/ZnO or bilayer of ZnO and Ba(OH)_2_ [ZnO/Ba(OH)_2_] or nanocomposite of ZnO and Ba(OH)_2_ [ZnO:Ba(OH)_2_] nanocomposites (40 nm)/PTB7-Th:PCBM(100 nm)/MoO_3_ (10 nm)/Ag (100 nm). Our study reveals that the performance of the devices with ZnO/Ba(OH)_2_ and ZnO:Ba(OH)_2_ nanocomposite as ETL supersedes that of devices with only ZnO as ETL. The plausible reasons for the improved performance of these devices are identified using contact angle measurements, atomic force microscopy (AFM), X-ray photoelectron spectroscopy (XPS), ultraviolet photoelectron spectroscopy (UPS) and photo-EIS measurements.

## 2. Experimental

### 2.1 Preparation of sol gel ZnO

Sol gel ZnO was prepared by the method reported in our previous work.^39^ At first, equimolar (0.25 M) solution of zinc acetate dihydrate (Aldrich, 99.9%) and ethanolamine was prepared in 10 ml of 2-methoxy ethanol. Thereafter, solution was allowed to stir rigorously for 12 h at 70 °C to get a homogeneous, clear and transparent solution. Thereafter solution was aged for 24 h before using it for spin-coating as an electron transport layer.

### 2.2 Preparation of barium hydroxide solution

The barium hydroxide solution was prepared by dissolving barium hydroxide in 2-methoxyethanol (2 mg ml^−1^).^26,38^ The Ba(OH)_2_ solution was filtered by a nylon filter with a diameter of 0.45 μm prior to the spin-cast.

### 2.3 Preparation of zinc oxide/barium hydroxide nanocomposites

For preparation of ZnO:Ba(OH)_2_ nanocomposite, ZnO stock solution was mixed with a set amount of Ba(OH)_2_ solution (3, 6, 9 and 12 w/w%) and stirred vigorously for 12 h. As obtained solutions were filtered by a nylon filter with a diameter of 0.45 μm prior to the spin-cast.

### 2.4 Device fabrication

**Fig. 1:**
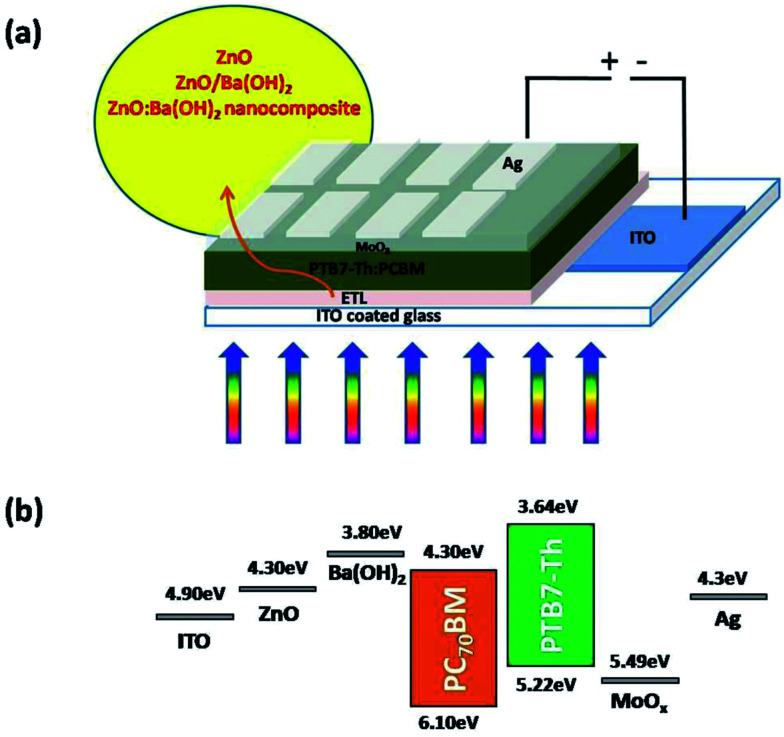
(a) Schematic of PTB7-Th:PCBM OSCs in an inverted geometry with ZnO, ZnO/Ba(OH)_2_ and ZnO:Ba(OH)_2_ nanocomposite as ETLs (b) energy levels of materials used in the solar cell device.

Devices were fabricated following the device architecture mentioned in the Fig. 1(a) and corresponding energy levels of the materials used in the fabrication are shown in the Fig. 1(b).^38,39^ At first, photoactive blend comprised of PTB7-Th (10 mg) and PCBM (15 mg) was prepared in 1 ml of DCB with 3% v/v of 1,8-diiodooctane (DIO) solvent as an additive. The solution was allowed to stir at 70 °C in the dark for at least 12 h. For the device fabrication, ITO coated glass substrates (Luminescence Technology corp. Taiwan, with a sheet resistance of 15 Ω sq^−1^ and transmittance > 85%) were cleaned by process reported in our previous work.^7,40^ For the devices with ZnO and ZnO:Ba(OH)_2_ nanocomposite as an ETL, ZnO and ZnO:Ba(OH)_2_ nanocomposite solutions which was previously synthesized, was spin-coated on pre-cleaned ITO substrates at 2000 rpm for 30 s to obtain a film of ∼40 nm. The substrates were then annealed at 200 °C for 15 min to remove excess solvents. Thereafter, substrates were transferred inside nitrogen filled glove box and a photoactive blend was spin-coated at 1100 rpm for 120 s. The films were allowed to get dried for 2 h and then subsequently, the devices were evaporated using the shadow mask by thermal evaporation of 10 nm MoO_3_ layer as a hole transport layer and 100 nm Ag electrode as anode. For the devices with ZnO/Ba(OH)_2_, an additional layer of Ba(OH)_2_ was spin-coated on the top of ZnO layer and allowed to dry before spin-coating the photoactive layer. Thereafter the film is annealed at 200 °C for 5 min to remove excess solvent if any.

### 2.5 Device characterization and testing

The current density–voltage (*J*–*V*) characteristics curves of fabricated devices were measured using a Keithley 2600 source meter and a Newport solar simulator (model number 91160) shines light with AM 1.5 G spectral distribution, which was calibrated using a certified reference solar cell to an intensity of 1000 W m^−2^. External quantum efficiency (EQE) spectra were obtained from Bentham's PVE300. The test cells were masked with a single aperture and care has been taken to exclude all light from entering the cell elsewhere, including shading of the edges of the substrates. The active device area is defined as 4.5 mm^2^ by using a shadow mask. Conductivity measurements of various ETLs were performed by Broadband Dielectric Spectroscopy at room temperature using Alpha-A high performance frequency analyser.

## 3. Results and discussion

**Fig. 2:**
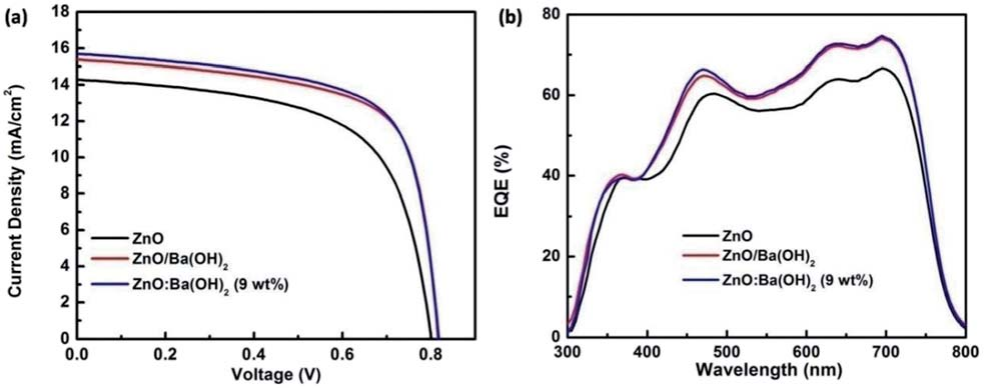
(a) *J*–*V* characteristics under illumination (AM 1.5 G, one sun) and (b) EQE spectra of devices with ZnO, ZnO/Ba(OH)_2_ and ZnO:Ba(OH)_2_ nanocomposite as an ETLs.

Fig. 2(a) and (b) presents the *J*–*V* characteristics curves and external quantum efficiency (EQE) spectra of devices with different ETLs. Photovoltaic parameters such as open circuit voltage (*V*_oc_), short-circuit current density (*J*_sc_), fill factor (FF), and PCE for the devices with ZnO, ZnO/Ba(OH)_2_ and ZnO:Ba(OH)_2_ (9 wt%) nanocomposite as an ETL are shown in Table 1 and for all the fabricated devices are summarized in Table S1.† As can be seen, for the control device with only ZnO as an ETL, *V*_oc_, *J*_sc_, FF and PCE are 803 mV, 14.28 mA cm^−2^, 62.30% and 7.12% respectively. However, for the devices with ZnO/Ba(OH)_2_ and ZnO:Ba(OH)_2_ nanocomposite as an ETL, improvement in performance parameters are noticed. As a result, devices with ZnO/Ba(OH)_2_ and ZnO:Ba(OH)_2_ nanocomposite as an ETL demonstrates PCE of 8.54 and 8.66% respectively. This is roughly 20% increase when compared to the devices with only ZnO as an ETL. Although there is a slight change in the *V*_oc_, it can be observed that the increase in the PCE mainly arises mainly due to increase in the *J*_sc_ and FF for devices with ZnO/Ba(OH)_2_ and ZnO:Ba(OH)_2_ nanocomposite as an ETL. Increase in the *J*_sc_ is also confirmed from the EQE measurements. *J*_sc_ calculated from the EQE spectra of the three devices are shown in the Table 1 and for all the fabricated devices in Table S1.† Maximum EQE of ∼70% is achieved in the case of devices with ZnO/Ba(OH)_2_ and ZnO:Ba(OH)_2_ (9 wt%) nanocomposite as an ETL as against ∼62% for the devices with only ZnO as an ETL. To investigate the plausible reasons for the improved performance and performance parameters like *V*_oc_, *J*_sc_, FF of devices with ZnO/Ba(OH)_2_ and ZnO:Ba(OH)_2_ (9 wt%) nanocomposite as an ETL, morphological studies, contact angle measurements, XPS, UPS and photo-EIS measurements are carried out and has been discussed below.

**Table 1:** Performance parameters for devices with ZnO, ZnO/Ba(OH)_2_ and ZnO:Ba(OH)_2_ nanocomposite as an ETLs

Electron transport layer (ETL)	*V*_oc_ (mV)	*J*_sc_ a (mA cm^−2^)	FF (%)	PCE^b^ (%)
ZnO	803	14.28 (14.05)	62.30	7.12 (7.07)
ZnO/Ba(OH)_2_	814	15.34 (15.56)	68.20	8.54 (8.46)
ZnO:Ba(OH)_2_ (9 wt%)	818	15.69 (15.70)	67.60	8.66 (8.59)

a *J*_sc_ as calculated from EQE is shown in parentheses.

b The average PCE is shown in parentheses. Average PCE was calculated using the results of 5 devices.

**Fig. 3:**
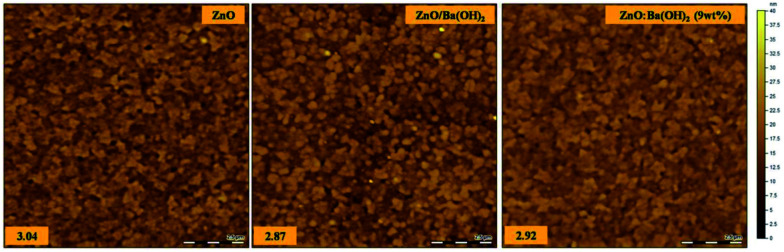
Atomic Force Microscopy (AFM) images of ZnO, ZnO/Ba(OH)_2_ and ZnO:Ba(OH)_2_ (9 wt%) nanocomposite thin films deposited on ITO coated glass substrates.

From the previous studies, it is well understood that better hydrophobic properties of ETLs are essential for the intimate contact with the deposited photoactive layer which is spin-coated from dispersion in an organic solvent.^41,42^ Thus, contact angle measurements were carried out with water drop as the probe for the surface of ZnO, ZnO/Ba(OH)_2_ and ZnO:Ba(OH)_2_ (9 wt%) nanocomposite and subsequent values are shown in Table 2 and for all the all the films studies, contact angle values are summarized in Table S2.† From both these tables, two observations were made: (1) it can be seen that in both cases *i.e.* ZnO/Ba(OH)_2_ and for all the composition of ZnO:Ba(OH)_2_ nanocomposite, contact angle is more than ZnO (2) it is observed that contact angle increases with the increase in the wt% of Ba(OH)_2_ in ZnO:Ba(OH)_2_ nanocomposite and saturates at around ∼52° for all the compositions after 6 wt%. From both these observations, it is confirmed that films of ZnO/Ba(OH)_2_ and ZnO:Ba(OH)_2_ nanocomposite are somewhat more hydrophobic than that of ZnO. But this small increment in contact angle for the films of ZnO/Ba(OH)_2_ and ZnO:Ba(OH)_2_ nanocomposite could not be the only reason for the improved PCE and therefore role of morphology and surface topography of ETL is investigated thereafter. It has been well known that the interfacial morphology and surface topography of ETL plays a crucial role and can significantly influence the device performance.^43,44^ Surface morphology and RMS surface roughness of ZnO, ZnO/Ba(OH)_2_ and ZnO:Ba(OH)_2_ (9 wt%) nanocomposite thin films deposited on ITO coated glass substrates are shown in the Fig. 3. It was observed that although the morphology of all the three films are quite similar but RMS surface roughness of the ZnO/Ba(OH)_2_ and ZnO:Ba(OH)_2_ (9 wt%) nanocomposite thin films are slightly less as compared to the ZnO. Thus, we believe that smooth surface of these film facilitates with slightly higher contact angle might be facilitating uniform, conformal and intimate contact with the PTB7-Th:PCBM photoactive layer therefore devices with ZnO/Ba(OH)_2_ and ZnO:Ba(OH)_2_ (9 wt%) nanocomposite thin films as an ETL performs better than devices with only ZnO as an ETL.

**Table 2:** Contact angle of the surface of films of ZnO, ZnO/Ba(OH)_2_ and ZnO:Ba(OH)_2_ nanocomposite thin films deposited on ITO coated glass substrates

Electron transport layer (ETL)	Contact angle (in deg)
ZnO	46.88 ± 0.65
ZnO/Ba(OH)_2_	49.59 ± 0.54
ZnO:Ba(OH)_2_ (9 wt%)	52.65 ± 0.81

**Fig. 4:**
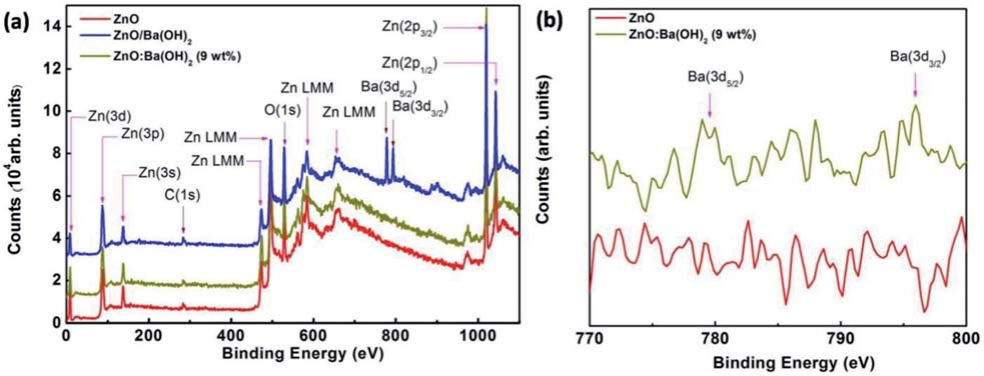
(a) XPS Survey scan spectra of ZnO, ZnO/Ba(OH)_2_ and ZnO:Ba(OH)_2_ (9 wt%) nanocomposite thin films deposited on ITO coated glass substrates. (b) XPS core level spectra of ZnO and ZnO:Ba(OH)_2_ (9 wt%) nanocomposite.

To confirm and demonstrate the presence of Ba in ZnO/Ba(OH)_2_ and ZnO:Ba(OH)_2_ (9 wt%) nanocomposite, XPS survey spectra of ZnO, ZnO/Ba(OH)_2_ and ZnO:Ba(OH)_2_ (9 wt%) nanocomposite acquired in the range of 0–1170 eV have been taken and are illustrated in Fig. 4(a). During photoemission studies, specimen charging was observed which was later calibrated by assigning the C 1s signal at 284.6 eV. To get the clean surface Ar ion sputtering performed on all three samples for 20 min with average base pressure maintained 6 × 10^−8^ torr. Survey spectra of Fig. 4(a) show sharp peaks of C 1s (284.6 eV), O 1s (531 eV) along with the expected peaks of Zn and Ba. Auger peak for Zn (LMM) was also observed. For ZnO, ZnO/Ba(OH)_2_ and ZnO:Ba(OH)_2_ (9 wt%) nanocomposite, two prominent peaks at ∼1021.75 eV and ∼1044.25 eV can be attributed to Zn (2p_3/2_) & Zn (2p_1/2_), which confirms the presence of ZnO phase formation.^23^ Also, for ZnO/Ba(OH)_2_, two prominent peaks at ∼781 eV and ∼796 eV can be attributed to Ba (3d_5/2_) & Zn (3d_3/2_).^45^ However, the intensity of these peaks decreases significantly in the case of ZnO:Ba(OH)_2_ (9 wt%) nanocomposite and these peaks are absent in the case of ZnO as can be seen in the Fig. 4(b). Thus, it is confirmed that Ba exist in both ZnO/Ba(OH)_2_ and ZnO:Ba(OH)_2_ (9 wt%) nanocomposite.

**Fig. 5:**
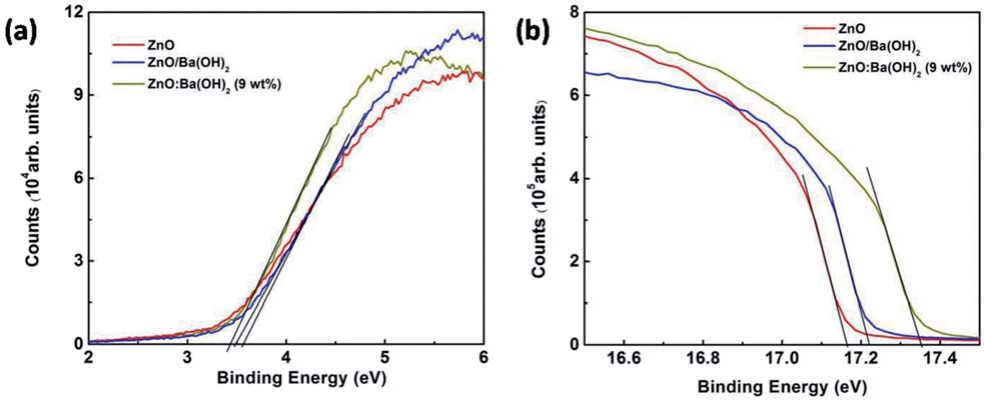
UPS spectra of ZnO, ZnO/Ba(OH)_2_ and ZnO:Ba(OH)_2_ (9 wt%) nanocomposite thin films deposited on ITO coated glass substrates (a) valence band region (b) cut-off region.

As discussed in the previous section of this paper, the increase in the PCE for devices with ZnO/Ba(OH)_2_ and ZnO:Ba(OH)_2_ nanocomposite as an ETL is mainly due to increase in the *J*_sc_ and FF. It is well known from the previous studies that *J*_sc_ depends on the photon absorption which is material property of donor polymer and both *J*_sc_ and FF depends on intrinsic mobility of donor and acceptor, morphology of photoactive and buffer layers and energy level of buffer layers used.^46^ In the present study, photon absorption, intrinsic mobility of donor and acceptor, morphology of photoactive layer must be same for the three devices as same photoactive layer comprised of PTB7-Th:PCBM has been employed. Further, as discussed in the previous section, there is a slight difference in the morphology, surface roughness and hydrophobic properties of films of ZnO, ZnO/Ba(OH)_2_ and ZnO:Ba(OH)_2_ nanocomposite. Thus, it is presumed that there must be the change in the energy levels of these films which is responsible for the increase in the PCE of devices with ZnO/Ba(OH)_2_ and ZnO:Ba(OH)_2_ nanocomposite as an ETL. To verify this presumption, UPS measurements are carried out to study the surface electronic energy levels ZnO, ZnO/Ba(OH)_2_ and ZnO:Ba(OH)_2_ (9 wt%) nanocomposite thin films and results are shown in Fig. 5(a) and (b). The HOMO level of ZnO, ZnO/Ba(OH)_2_ and ZnO:Ba(OH)_2_ (9 wt%) nanocomposite thin films are found to be 7.58, 7.52 and 7.28 eV respectively. This trend is similar to the trend reported for previously reported different ZnO based nanocomposite.^34^ Thus, it is clear that HOMO level of the ZnO/Ba(OH)_2_ and ZnO:Ba(OH)_2_ nanocomposite thin films are lower than that of ZnO (Table 3). The HOMO level was determined by the eqn (1) mentioned in the previous studies:^20,47^

1 HOMO level = *hν* − (*E*_1_ − *E*_2_)

**Table 3:** Various energy levels of ZnO, ZnO/Ba(OH)_2_ and ZnO:Ba(OH)_2_ (9 wt%) nanocomposite thin films deposited on ITO coated glass substrates^a^

Electron transport layer (ETL)	*E*_1_	*E*_2_	Δ*E*	HOMO	*E*_g_	LUMO
ZnO	17.16	3.53	13.63	7.58	3.22	4.36
ZnO/Ba(OH)_2_	17.22	3.53	13.66	7.52	3.29	4.23
ZnO:Ba(OH)_2_ (9 wt%)	17.36	3.43	13.93	7.28	3.34	3.94

a *E*_1_ is upper emission onset energies and *E*_2_ is the lower emission onset energies.

Here, *hν* is incident photon energy (21.2 eV) of He I, and values of *E*_1_ and *E*_2_ are for ZnO, ZnO/Ba(OH)_2_ and ZnO:Ba(OH)_2_ (9 wt%) nanocomposite thin films are shown in Table 3.^33,47^ The *E*_1_ was determined by linear extrapolation to zero at the yield of secondary electrons, and the *E*_2_ is the onset relative to the Fermi level (*E*_f_) of Au (0 eV), where the *E*_f_ is determined from the Au substrate.^47^ The LUMO level of ZnO, ZnO/Ba(OH)_2_ and ZnO:Ba(OH)_2_ (9 wt%) nanocomposite thin films is determined by using eqn (2) mentioned in the previous studies:^47^

2 LUMO = HOMO − optical band gap,

The LUMO levels of ZnO, ZnO/Ba(OH)_2_ and ZnO:Ba(OH)_2_ (9 wt%) nanocomposite thin films are found to be 4.36, 4.26 and 3.94 eV respectively. Thus, it is evident that the LUMO of the ZnO/Ba(OH)_2_ and ZnO:Ba(OH)_2_ (9 wt%) nanocomposite thin films is smaller than that of ZnO. This trend is expected and can be explained based on the mechanism reported in the previous studies in which they have discussed the formation of a low work function Zn–O–Ba complex.^26,48^ Their study suggests that a thin layer of O–Ba is formed on the ZnO surface with Ba ions pointing upwards. Further, the formation of O–Ba replaces the hydroxyl group on the ZnO surface by surface chemical reaction: ^−^OH + Ba^+^ → O–Ba + H^+^, a phenomenon similar to chemisorption of Cl-terminated molecules onto ITO. The interface dipoles with a negative charge toward ZnO and the corresponding positive charge toward the upward direction result in the lowering of the vacuum level of ZnO and this might be facilitating the charge transport from PCBM to ETLs.^21,48^ Further, variation in conductivities of the ETLs are given in Fig. S1b of ESI† and device performance of various devices was compared with the intensity dependent photo-EIS measurements and has been discussed in the subsequent section.

**Fig. 6:**
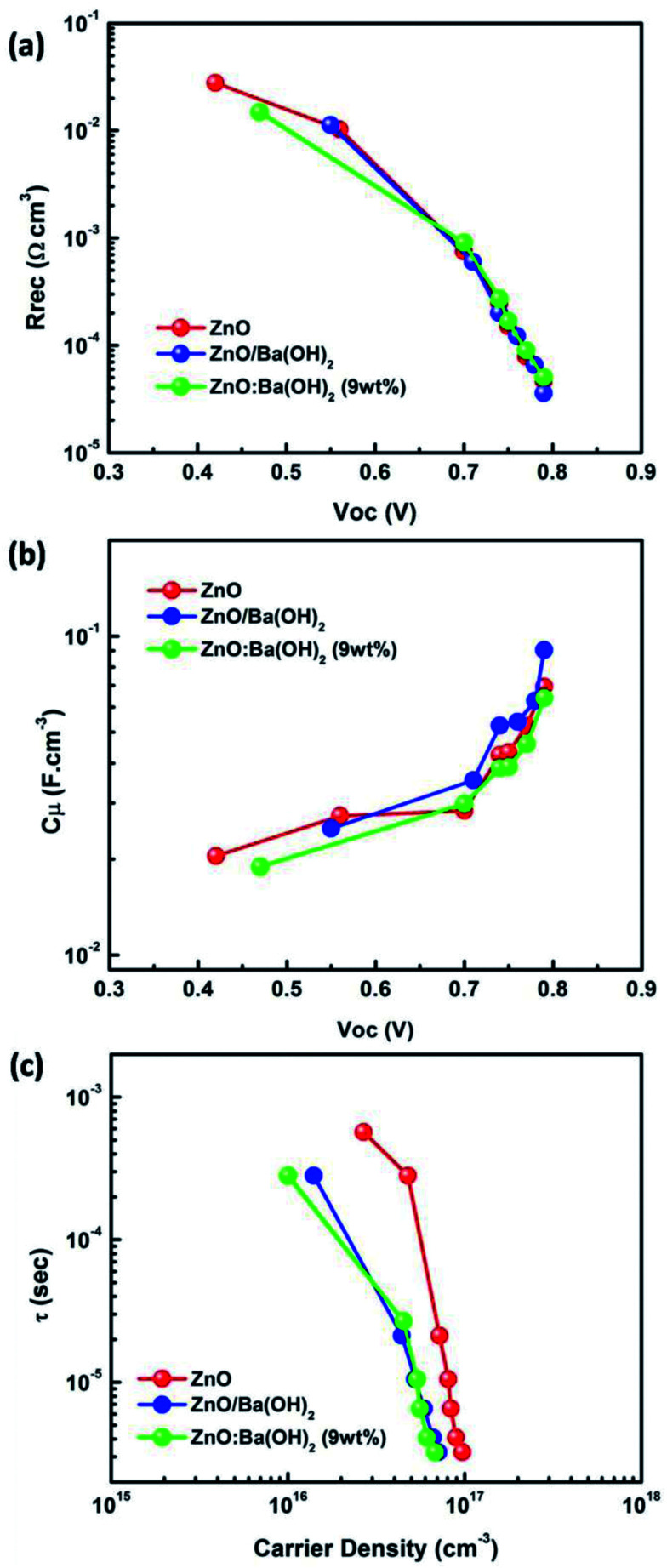
(a) Recombination resistance (*R*_rec_) as a function of *V*_oc_ (b) chemical capacitance (*C*_μ_) as a function of *V*_oc_ (c) carrier lifetime as a function of the photo-generated carrier density for PTB7-Th:PCBM OSCs with ZnO, ZnO/Ba(OH)_2_ and ZnO:Ba(OH)_2_ nanocomposite as an ETLs.

Fig. S3† illustrates Nyquist plot (both raw and fitted) of PTB7-Th:PCBM OSCs with ZnO, ZnO/Ba(OH)_2_ and ZnO:Ba(OH)_2_ nanocomposite as ETLs under different illumination intensity. It can be clearly seen that for all the three ETLs, photo-EIS spectra as shown in the Fig. S3(a–c)† consist of only one semi-circle. This is in agreement with the previous reports of PTB7-Th:PCBM OSC and can be explained on the basis of molecular packaging of donor polymer.^49,50^ In the case of PTB7-Th, like other low bandgap donor polymers, it is a face-on orientation making the inter-electrode transport proceeds through the π–π stacking. As a result, the second semicircle which is generally observed in P3HT:PCBM OSC and corresponds to the charge transport is absent.^6,8,9^ Therefore, Nyquist plot for PTB7-Th:PCBM OSC can be fitted by a simple circuit shown in the Fig. S4† which consists of a recombination resistance (*R*_rec_) and the chemical capacitance (*C*_μ_) along with the series resistance due to contact and wires (*R*_s_). The obtained values of resistance and the capacitance after fitting were plotted as a function of *V*_oc_ and have been shown in the Fig. 6(a) and (b) respectively. A critical investigation of these curves approves an exponential decrease and increase of resistance and capacitance respectively with the increase in *V*_oc_. Thus, it can be concluded that they represents a bulk and interfacial recombination resistance and chemical capacitance.^40,51^ A meaningful analysis of charge recombination processes within the device can be obtained by fitting the recombination resistance and chemical capacitance as a function of *V*_oc_ using the exponential laws mentioned in the eqn (3) and (4).^52^ The obtained values of *α* and *β* can be seen the Table S3.† Further, the response time representative of the recombination processes is calculated from the characteristic frequency (*ω*) at the top of the arc, where 2π*ω* = 1/*τ*.^53^ For all the devices with different ETLs, effective recombination times (*τ*) range in the order of 10^−4^ to 10^−6^ s. Additionally, as discussed in previous studies, it is feasible to estimate the charger carrier density (*n*) from the chemical capacitance (*C*_μ_) using eqn (5).^52^

3 *R*_rec_ ∝ exp(−*βV*_oc_/*κ*_B_*T*)

4 *C*_μ_ ∝ exp(*αV*_oc_/*κ*_B_*T*)

5
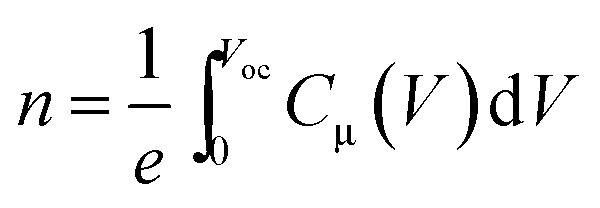


Fig. 6(c) illustrates the carrier lifetime as a function of the photo-generated carrier density. All the curves show decay dynamics that follow a power law trend mentioned in the eqn (6).^51^ The obtained value of *λ* for PTB7-Th:PCBM OSCs with ZnO, ZnO/Ba(OH)_2_ and ZnO:Ba(OH)_2_ nanocomposite as ETLs are shown in the Table S3.† It is necessary to mention here that the impedance measurements allow to establish a direct relation between the exponents of the capacitance and resistance dependences on *V*_oc_, and the power-law exponent of *τ*(*n*) function as mentioned in the eqn (7).^51,52^ The obtained values of *λ* using the eqn (7) for PTB7-Th:PCBM OSCs with ZnO, ZnO/Ba(OH)_2_ and ZnO:Ba(OH)_2_ nanocomposite as ETLs are also shown in the Table S3.†

6 *τ* ∝ *n*^−*λ*^

7
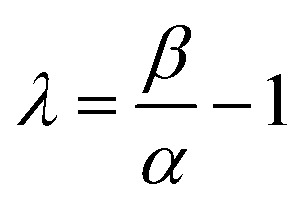


It can be clearly observed that values of *λ* as obtained from eqn (6) and (7) for the PTB7-Th:PCBM OSCs with ZnO/Ba(OH)_2_ and ZnO:Ba(OH)_2_ nanocomposite as ETLs are in close agreement suggesting that order of recombination (1 + *λ*) depends not only on the features of the density of states but also on the flux of carrier loss through the parameter *β*. However, the values of *λ* as obtained from eqn (6) and (7) for the PTB7-Th:PCBM OSCs with ZnO as ETL are different. In this case, the similar value of *λ* (as calculated using eqn (7)) can be obtained by using the relation *λ* = 1/*α* thereby confirming that order of the recombination exclusively depends on the energy distribution of the trapped states.^52,54^ This is because the above analyses recognize the recombination as a multiple trapping mechanism in which trapped holes can either recombine with electrons at the acceptor levels or be retrapped at an exponential tail of donor states.^52,54^ Thus, photo-EIS measurements confirms that PTB7-Th:PCBM OSCs with ZnO/Ba(OH)_2_ and ZnO:Ba(OH)_2_ nanocomposite as ETLs performs better than that of PTB7-Th:PCBM OSCs with ZnO as an ETL because the presence of Ba(OH)_2_ restricts the multiple trapping and minimizes the recombination which was otherwise observed in devices with only ZnO as an ETL.^52^

## 4. Conclusion

In conclusion, we have compared the device performance of PTB7-Th:PCBM OSCs in an inverted geometry with ZnO, ZnO/Ba(OH)_2_ and ZnO:Ba(OH)_2_ nanocomposite as an ETLs. Our study confirms that devices with ZnO/Ba(OH)_2_ and ZnO:Ba(OH)_2_ nanocomposite as ETL demonstrates improved performance parameters compared to devices with only ZnO as ETL. It is observed that films of ZnO/Ba(OH)_2_ and ZnO:Ba(OH)_2_ nanocomposite have low work function and are slightly more smooth and hydrophobic as compared to ZnO films. This might have suppressed the charge recombination and thereby improved the charge collection as has been confirmed by EIS measurements.

## Conflicts of interest

There are no conflicts to declare.

## Supplementary Material

RA-008-C7RA13428B-s001
