# Supplementary material for: Interface engineering through electron transport layer modification for high efficiency organic solar cells
Source: RSC Adv. 2018 Feb 5;8(11):5984–91. doi: 10.1039/c7ra13428b (PMC9078166; doi:10.1039/c7ra13428b)
Supplement: RA-008-C7RA13428B-s001 [file RA-008-C7RA13428B-s001.pdf]

Electronic Supplementary Material (ESI) for RSC Advances.
This journal is © The Royal Society of Chemistry 2018

# Supplementary Information

## Interface engineering through electron transport layer modification for high efficiency organic solar cells

Kunal Borse [a,b,1], Ramakant Sharma [a,1], Dipti Gupta [a,*] and Aswani Yella [a,*]

[a] Department of Metallurgical Engineering and Materials Science,
Indian Institute of Technology Bombay, Powai, Mumbai- 400076, India.

[b] Department of Metallurgy, Government Polytechnic, Kolhapur- 416004, India

* Corresponding Author Email: diptig@iitb.ac.in (D.G), aswani.yella@iitb.ac.in (A.Y)

[1] Kunal Borse and Ramakant Sharma contributed equally to this work.

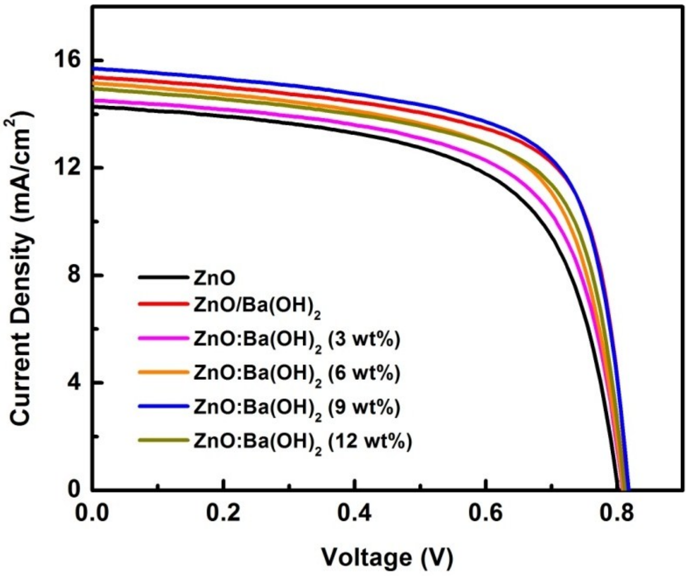


Figure S1 *J*-*V* characteristics curves under illumination (AM 1.5G, one sun) of devices with ZnO, $ZnO/Ba(OH)_2$ and $ZnO:Ba(OH)_2$ nanocomposite as an ETLs.

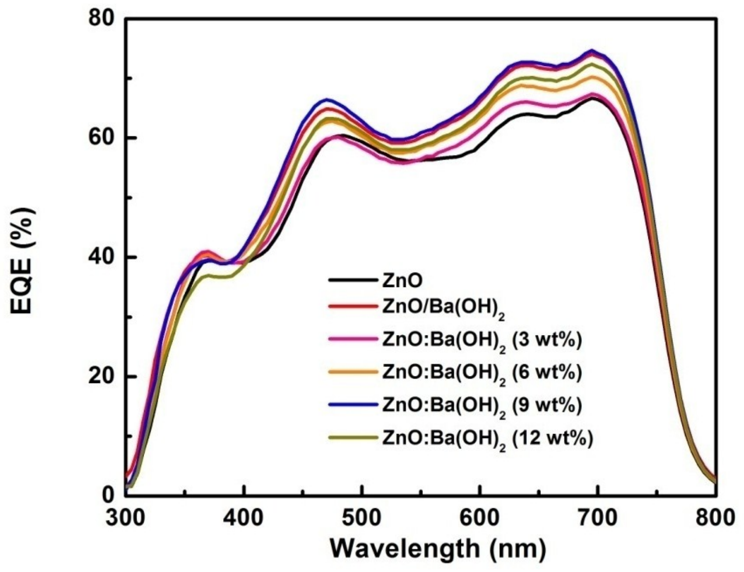


Figure S2 EQE spectra of devices with ZnO, $ZnO/Ba(OH)_2$ and $ZnO:Ba(OH)_2$ nanocomposite as an ETLs.

Table S1 Performance parameters for devices with ZnO, $ZnO/Ba(OH)_2$ and $ZnO:Ba(OH)_2$ nanocomposite as an ETLs.

| Electron Transport Layer (ETL) | $V_{oc}$ (mV) | $J_{sc}$[a] (mA/cm$^2$) | FF (%) | PCE[b] (%) |
|---|---|---|---|---|
| ZnO | 803 | 14.28 (14.05) | 62.30 | 7.12 (7.07) |
| $ZnO/Ba(OH)_2$ | 814 | 15.34 (15.56) | 68.20 | 8.54 (8.46) |
| $ZnO:Ba(OH)_2$ (3wt%) | 803 | 14.50 (14.33) | 63.90 | 7.44 (7.39) |
| $ZnO:Ba(OH)_2$ (6wt%) | 810 | 15.15 (14.90) | 65.10 | 7.98 (7.92) |
| $ZnO:Ba(OH)_2$ (9wt%) | 818 | 15.69 (15.70) | 67.60 | 8.66 (8.59) |
| $ZnO:Ba(OH)_2$ (12wt%) | 813 | 14.95 (15.07) | 66.60 | 8.07 (8.01) |

[a] Jsc as calculated from EQE is shown in parentheses. [b] The average PCE is shown in parentheses. Average PCE was calculated using the results of 5 devices.

Table S1a Comparison of performance parameters for PTB7-Th: PCBM devices with ZnO, $ZnO/Ba(OH)_2$ and $ZnO:Ba(OH)_2$ nanocomposite as an ETLs with the previously reported similar studies.

| Work | Cathode configuration | Anode Configuration | $V_{oc}$ (mV) | $J_{sc}$[a] (mA/cm$^2$) | FF (%) | PCE[b] (%) |
|---|---|---|---|---|---|---|
| Present | ITO/ $ZnO/Ba(OH)_2$ | $MoO_3$/Ag | 814 | 15.34 (15.56) | 68.20 | 8.54 (8.46) |
| Present | ITO/ $ZnO:Ba(OH)_2$ (9wt%) | $MoO_3$/Ag | 818 | 15.69 (15.70) | 67.60 | 8.66 (8.59) |
| Previous[1] | ITO/patterned ZnO | $MoO_3$/Al | 780 | 19.47 | 66.90 | 10.10 |
| Previous[2] | ITO/IZO | $MoO_3$/Ag | 790 | 16.42 | 70.2 | 9.11 |
| Previous[3] | ITO/AZO | $MoO_3$/Ag | 800 | 17.70 | 70.70 | 9.94 |
| Previous[4] | ITO/PFN | $MoO_3$/Al | 830 | 17.43 | 73.80 | 10.61 |
| Previous[5] | ITO/C 60 –SB | $MoO_3$/Ag | 750 | 18.24 | 66.00 | 9.08 |
| Previous[6] | ITO/ZnO:PBI-H | $MoO_3$/Al | 820 | 17.69 | 72.90 | 10.59 |
| Previous[7] | ITO/ZnO/[BMIM] BF 4 | $MoO_3$/Ag | 780 | 17.70 | 73.50 | 10.15 |
| Previous[8] | ITO/ZnO–C 60 | $MoO_3$/Ag | 800 | 15.73 | 74.30 | 9.35 |

[a] Jsc as calculated from EQE is shown in parentheses. [b] The average PCE is shown in parentheses. Average PCE was calculated using the results of 5 devices.

Table S1b Conductivities of ZnO, ZnO/$Ba(OH)_2$ and ZnO:$Ba(OH)_2$ nanocomposite thin films

| Electron Transport Layer (ETL) | Conductivity (S/cm) |
|---|---|
| ZnO | $51.9 \times 10^{-3}$ |
| ZnO/$Ba(OH)_2$ | $91.4 \times 10^{-3}$ |
| ZnO:$Ba(OH)_2$ (3wt%) | $74.8 \times 10^{-3}$ |
| ZnO:$Ba(OH)_2$ (6wt%) | $75.2 \times 10^{-3}$ |
| ZnO:$Ba(OH)_2$ (9wt%) | $93.2 \times 10^{-3}$ |
| ZnO:$Ba(OH)_2$ (12wt%) | $89.3 \times 10^{-3}$ |

Table S2 Contact angle of the surface of films of ZnO, ZnO/$Ba(OH)_2$ and ZnO:$Ba(OH)_2$ nanocomposite thin films deposited on ITO coated glass substrates.

| Electron Transport Layer (ETL) | Contact Angle (in deg) |
|---|---|
| ZnO | 46.88 ± 0.65 |
| ZnO/$Ba(OH)_2$ | 49.59 ± 0.54 |
| ZnO:$Ba(OH)_2$ (3wt%) | 47.98± 0.60 |
| ZnO:$Ba(OH)_2$ (6wt%) | 52.09 ± 1.59 |
| ZnO:$Ba(OH)_2$ (9wt%) | 52.65 ± 0.81 |
| ZnO:$Ba(OH)_2$ (12wt%) | 51.75 ± 0.87 |

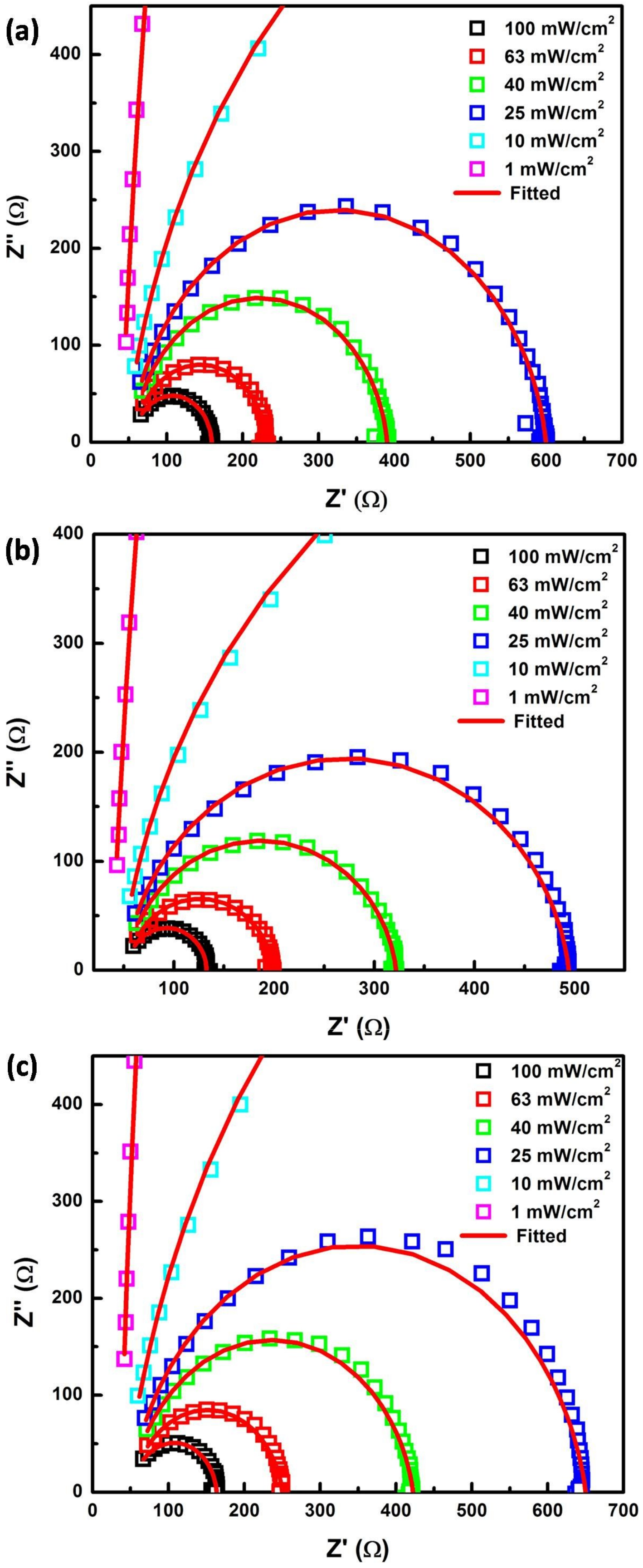


Figure S3 Nyquist plots of PTB7-Th:$PC_{70}BM$ OSCs with (a) ZnO (b) ZnO/$Ba(OH)_2$ and (c) ZnO:$Ba(OH)_2$ nanocomposite as an ETLs under different white light illumination intensities

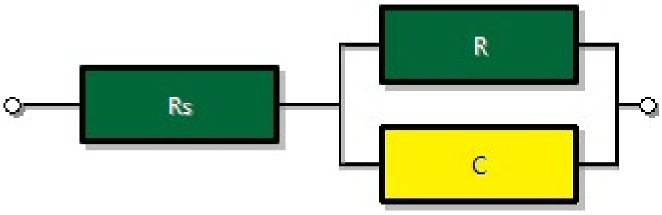


Figure S4 Equivalent circuit used for fitting for different impedance curves.

Table S3 Order of recombination for devices with ZnO, ZnO/$Ba(OH)_2$ and ZnO:$Ba(OH)_2$ nanocomposite as an ETLs.

| Electron Transport Layer (ETL) | α | β | λ (from Fig 6 (c)) | λ (from Eq (7)) |
|---|---|---|---|---|
| ZnO | 0.29 | 0.70 | 3.93 | 1.36 |
| ZnO/$Ba(OH)_2$ | 0.27 | 0.86 | 2.75 | 2.12 |
| ZnO:$Ba(OH)_2$ (9 wt%) | 0.25 | 0.76 | 2.32 | 2.03 |

**References:**


1 J. De Chen, C. Cui, Y. Q. Li, L. Zhou, Q. D. Ou, C. Li, Y. Li and J. X. Tang, *Adv. Mater.*, 2015, 27, 1035–1041.

2 S. H. Liao, H. J. Jhuo, P. N. Yeh, Y. S. Cheng, Y. L. Li, Y. H. Lee, S. Sharma and S. A. Chen, *Sci. Rep.*, 2014, 4, 4–10.

3 L. K. Jagadamma, M. Al-Senani, A. El-Labban, I. Gereige, G. O. Ngongang Ndjawa, J. C. D. Faria, T. Kim, K. Zhao, F. Cruciani, D. H. Anjum, M. A. McLachlan, P. M. Beaujuge and A. Amassian, *Adv. Energy Mater.*, 2015, 5, 1–12.

4 Z. He, B. Xiao, F. Liu, H. Wu, Y. Yang, S. Xiao, C. Wang, T. P. Russell and Y. Cao, *Nat. Photonics*, 2015, 9, 174–179.

5 Y. Liu, Z. Page, S. Ferdous, F. Liu, P. Kim, T. Emrick and T. Russell, *Adv. Energy Mater.*, 2015, 5, 1–6.

6 L. Nian, W. Zhang, N. Zhu, L. Liu, Z. Xie, H. Wu, F. Würthner and Y. Ma, *J. Am. Chem. Soc.*, 2015, 137, 6995–6998.

7 W. Yu, L. Huang, D. Yang, P. Fu, L. Zhou, J. Zhang and C. Li, *J. Mater. Chem. A*, 2015, 3, 10660–10665.

8 S. H. Liao, H. J. Jhuo, Y. S. Cheng and S. A. Chen, *Adv. Mater.*, 2013, 25, 4766–4771.
